# P-1103. In Vitro Activity of Zoliflodacin Against Baseline *Neisseria gonorrhoeae* Isolates from US Participants in a Global Phase 3 Randomized Controlled Trial

**DOI:** 10.1093/ofid/ofae631.1291

**Published:** 2025-01-29

**Authors:** Sarah McLeod, Varalakshmi Elango, Esther Bettiol, Alison Luckey

**Affiliations:** Innoviva Specialty Therapeutics, Inc., Waltham, Massachusetts; Global Antibiotic R&D Partnership, Chennai, Tamil Nadu, India; Global Antibiotic R&D Partnership, Chennai, Tamil Nadu, India; Global Antibiotic R&D Partnership (GARDP), Geneva, Geneve, Switzerland

## Abstract

**Background:**

Zoliflodacin is an oral, single-dose, first-in-class spiropyrimidinetrione bacterial topoisomerase inhibitor with activity against multidrug-resistant strains of *Neisseria gonorrhoeae*. Efficacy and safety of zoliflodacin for the treatment of uncomplicated gonorrhea was studied in a global Phase 3 trial that demonstrated non-inferiority to ceftriaxone plus azithromycin. In this study, the antibiotic susceptibility of baseline *N. gonorrhoeae* isolates from US participants in the Phase 3 trial was characterized.

**Figure:**
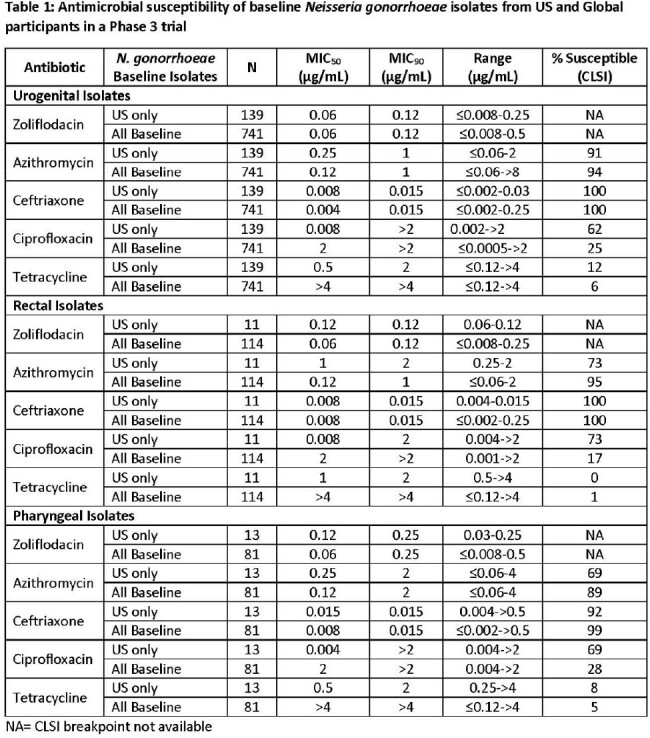

**Methods:**

Overall, 163 *N. gonorrhoeae* baseline isolates (139 urogenital, 13 pharyngeal, 11 rectal) were cultured from participants enrolled at 5 US clinical sites. Isolates were speciated using MALDI-TOF. MICs were performed using agar dilution according to CLSI guidelines. CLSI interpretive criteria were applied where available.

**Results:**

The zoliflodacin MIC_50/90_ was 0.06/0.12 µg/mL (MIC range ≤0.008 – 0.25 µg/mL) against 139 baseline urogenital *N. gonorrhoeae* isolates, which is consistent with that seen among pharyngeal and rectal US isolates and the global set of baseline isolates from the Phase 3 trial (Table 1). Azithromycin susceptibility rates were lower for the US isolates (69-91% depending on the body site of infection) compared to the global isolates (89-95%); however, susceptibility to ciprofloxacin was higher (62-73% vs 17-28%). For all isolates, susceptibility to tetracycline was low (0-12%) while ceftriaxone susceptibility was high ( >99%). There was 1 ceftriaxone resistant isolate from a US patient (MIC >0.5 µg/mL). This was a pharyngeal isolate also resistant to ciprofloxacin and tetracycline, but susceptible to azithromycin. Zoliflodacin demonstrated an MIC of 0.12 µg/mL against this isolate.

**Conclusion:**

Against *N. gonorrhoeae* baseline isolates from US participants from a Phase 3 trial for uncomplicated gonorrhea, the in vitro potency of zoliflodacin was consistent with that observed against the global set of baseline isolates and US surveillance studies. Zoliflodacin maintained activity against multidrug-resistant isolates. These data complement the recent Phase 3 results and support the continued development of zoliflodacin for patients with uncomplicated gonorrhea.

**Disclosures:**

**Sarah McLeod, PhD**, Innoviva Specialty Therapeutics: Employee|Innoviva Specialty Therapeutics: Stocks/Bonds (Public Company) **Alison Luckey, MD**, GARDP: Employee of GARDP, a non-profit Swiss-based foundation, which receives government and private-funded institutional grants/research support

